# Prediction of radiographic progression pattern in patients with ankylosing spondylitis using group-based trajectory modeling and decision trees

**DOI:** 10.3389/fmed.2022.994308

**Published:** 2022-10-20

**Authors:** Juyeon Kang, Tae-Han Lee, Seo Young Park, Seunghun Lee, Bon San Koo, Tae-Hwan Kim

**Affiliations:** ^1^Division of Rheumatology, Department of Internal Medicine, Inje University Busan Paik Hospital, Inje University College of Medicine, Busan, South Korea; ^2^Department of Internal Medicine, Kyungpook National University Chilgok Hospital, Daegu, South Korea; ^3^Department of Statistics and Data Science, Korea National Open University, Seoul, South Korea; ^4^Department of Radiology, Hanyang University College of Medicine, Hanyang University Seoul Hospital, Seoul, South Korea; ^5^Division of Rheumatology, Department of Internal Medicine, Inje University Seoul Paik Hospital, Inje University College of Medicine, Seoul, South Korea; ^6^Department of Rheumatology, Hanyang University Hospital for Rheumatic Diseases, Seoul, South Korea

**Keywords:** ankylosing spondylitis (AS), radiographic progression, trajectory modeling, decision tree, prediction

## Abstract

**Objective:**

This study aimed to identify trajectories of radiographic progression of the spine over time and use them, along with associated clinical factors, to develop a prediction model for patients with ankylosing spondylitis (AS).

**Methods:**

Data from the medical records of patients diagnosed with AS in a single center were extracted between 2001 and 2018. Modified Stoke Ankylosing Spondylitis Spinal Scores (mSASSS) were estimated from cervical and lumbar radiographs. Group-based trajectory modeling classified patients into trajectory subgroups using longitudinal mSASSS data. In multivariate analysis, significant clinical factors associated with trajectories were selected and used to develop a decision tree for prediction of radiographic progression. The most appropriate group for each patient was then predicted using decision tree analysis.

**Results:**

We identified three trajectory classes: class 1 had a uniformly increasing slope of mSASSS, class 2 showed sustained low mSASSS, and class 3 showed little change in the slope of mSASSS but highest mSASSS from time of diagnosis to after progression. In multivariate analysis for predictive factors, female sex, younger age at diagnosis, lack of eye involvement, presence of peripheral joint involvement, and low baseline erythrocyte sedimentation rate (log) were significantly associated with class 2. Class 3 was significantly associated with male sex, older age at diagnosis, presence of ocular involvement, and lack of peripheral joint involvement when compared with class 1. Six clinical factors from multivariate analysis were used for the decision tree for classifying patients into three trajectories of radiographic progression.

**Conclusion:**

We identified three patterns of radiographic progression over time and developed a decision tree based on clinical factors to classify patients according to their trajectories of radiographic progression. Clinically, this model holds promise for predicting prognosis in patients with AS.

## Introduction

Ankylosing spondylitis (AS) is a chronic immune-mediated arthritis characterized by progressive inflammation of the axial skeleton, peripheral joints, and entheses; and extra-articular manifestations such as uveitis and inflammatory bowel disease (1–4). In many patients, structural damage to the axial spine leads to decreased physical function and quality of life as well as permanent limitations to spinal mobility (5–7). Developing a method to predict the arthritic progression of the spine in AS would have a large impact, as this may allow clinicians to initiate earlier intervention to limit disability. Many previous studies have been conducted to predict the progression of AS, showing factors such as smoking, acute-phase reactant level, bath ankylosing spondylitis disease activity index (BASDAI), age, and educational status to be associated with disease progression (8–14). Disease states and structural damage may be heterogeneous over time owing to chronic progression and variability in clinical presentation. However, few studies have yet identified predictive patterns of disease progression over time for AS (15, 16).

Trajectory analysis is a method with the ability to predict patterns over time based on the assumption that individual variability will follow a specific pattern (17–19). The expected trajectory of each subgroup is based on repetitive observations over time, and the analysis assumes that the subgroups are part of the same population. However, all individuals are classified within given subgroups that share more intra-group similarities than those found inter-group (17, 19, 20). For this study, we applied group-based trajectory modeling (GBTM), a sub-concept of trajectory analysis and statistical approach designed to group longitudinal observations into interrelated subgroups.

Another method for prediction of disease progression is decision tree analysis, a classifying and predicting data mining technology successful for predicting and explaining the relationship between certain measured values and target values of an item (21, 22). This method is advantageous for fast construction and generation of easy-to-interpret decision rules, and thus is often used in prediction models for disease diagnosis and prognosis in medical fields (23–27).

The purpose of this study was to predict the radiographic progression of AS patients by identifying the progression trajectories for the spine and constructing a decision tree based on these trajectories. The measure of disease progression was analyzed using the modified Stoke Ankylosing Spondylitis Spinal Score (mSASSS), the most useful tool for evaluating radiographic damage to the spine in patients with AS (28).

## Materials and methods

### Data collection

We investigated the data of adult patients aged ≥18 at the time of the observation, who were diagnosed with AS and underwent outpatient care at a single tertiary hospital, Hanyang University Hospital for Rheumatic Diseases, between January 2001 and December 2018. Patients who met the 1984 modified New York criteria (29) were diagnosed with AS. Among these 1,280 patients, 1,125 with two or more sets of spinal radiographs were included. This study was approved by the institutional review board (IRB) of Hanyang University (HYUH 2021-10-013) and was performed in accordance with the principles of the Declaration of Helsinki and Good Clinical Practice guidelines. As our study design used only medical records and radiographs, the IRB waived the requirement for patient consent.

Data on demographic and clinical features were obtained from patient medical records including age, sex, disease duration, smoking history, human leukocyte antigen (HLA)-B27 status, history of ocular involvement, peripheral arthritis history, and serum erythrocyte sedimentation rate (ESR), C-reactive protein (CRP) levels at diagnosis, and initial BASDAI.

**FIGURE 1:**
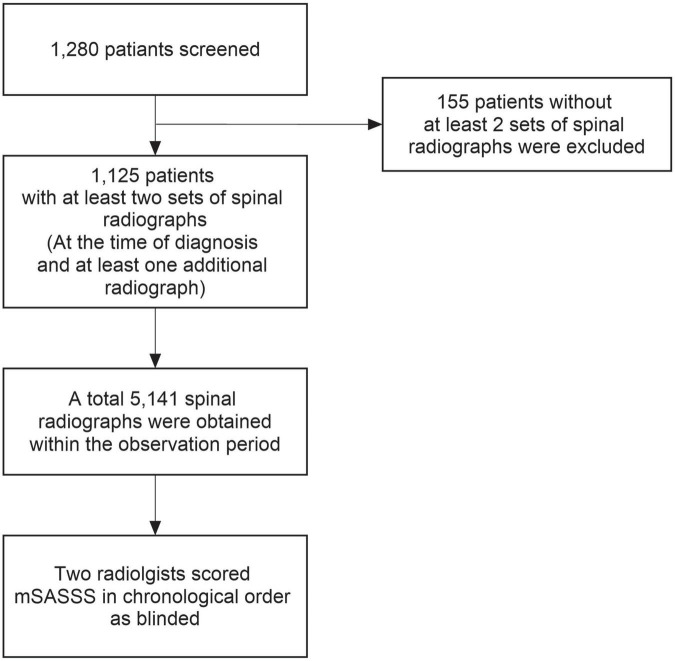
Flowchart of patient selection and radiographic data collection. mSASSS, modified Stoke Ankylosing Spondylitis Spinal Score.

Plane radiographs of the cervical and lumbar spine previously obtained at the outpatient clinic were analyzed. Since all radiographs were taken only by medical necessity, the duration between radiographs was different for each patient. Therefore, we analyzed the radiograph interval of each patient assuming that it was the same. During the entire follow-up, we obtained 5,141 spinal radiographs from 1,125 patients. The radiographs were scored in chronological order by two radiologists who were blinded to patient’s clinical information. After the scoring of radiographs was complete, accuracy of mSASSS was examined. Intra-observer reliability with consistency for each reader was calculated to be excellent (intraclass coefficient [ICC] = 0.978, 95% confidence interval [CI] = 0.976–0.979). Inter-observer reliability with agreement between two readers was also excellent (ICC = 0.946, 95% CI = 0.941–0.950) (30–32). The patient selection and radiographic data collection processes are shown in Figure 1.

### Statistical analysis

We used GBTM to identify the groups with differing mSASSS trajectories. Since mSASSS assessment time differed among patients, time since diagnosis was used as the time variable for the analysis. To determine the number of groups that best described the data, we employed commonly used metrics such as the Akaike information criteria (AIC), Bayesian information criteria (BIC), average posterior probability of assignment (APPA), odds of correct classification (OCC), and percentage of patients in the smallest group (17). Final choice on the number of classes was determined so that the model had the smallest AIC and BIC, reasonable APPA and OCC, and at least 10% of the total number of patients in the smallest group. We also used varying orders of polynomials, inclusion of random effects, and various assumptions of variance across time and groups; based on statistical fit and clinical plausibility, we selected the model with a second order polynomial, no random effects, and assumed equal variance across time and groups. We then further conducted comparisons of baseline characteristics among trajectory groups using analysis of variance or its non-parametric counterpart, the Kruskal–Wallis test, for continuous variables and the chi-square test for categorical variables. To identify predictors related to disease progression, two multivariate logistic regression models were fitted, since two binary classifiers were needed to classify patients into three trajectory groups.

Next, a decision tree based on clinical factors was identified to predict group membership identified through GBTM, and its accuracy was evaluated. We used the recursive partitioning for classification algorithm (33).

All statistical analyses were performed using R statistical language version 4.1.2, and *p*-values <0.05 were considered statistically significant.

## Results

**FIGURE 2:**
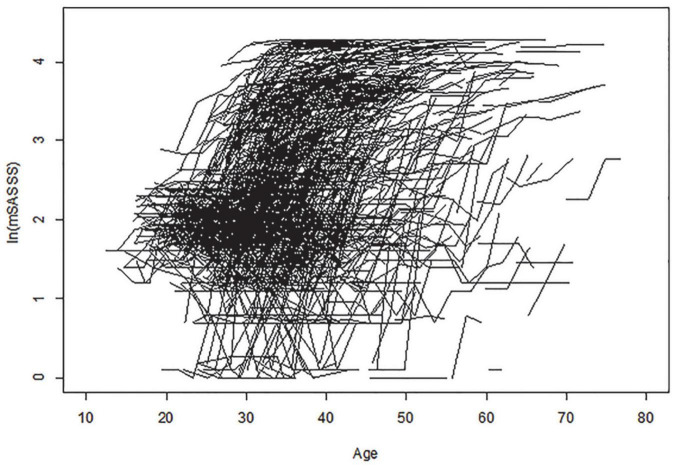
Change in individual patient mSASSS. Age is shown along the x-axis, and logarithmic transformed total mSASSS is shown along the y-axis. Each individual line represents a patient entry with the total mSASSS of at least two sets of radiographs. mSASSS, modified Stoke Ankylosing Spondylitis Spinal Score.

Among the 1,125 patients enrolled in this study, 995 (88.4%) were men, and mean age at diagnosis was 31.97 ± 9.40 years. In total, 1,081 (96.5%) patients were HLA-B27 positive. The average number (standard deviation [SD]) of spinal radiograph per patient during the study period was 4.6 (1.2) and follow-up period of 8.4 (2.9) years per patient, the median number of mSASSS per patient (min, max) was 4 (2, 8), and the average interval (SD) between mSASSS (spinal radiograph) was 2.4 (0.7) years. Table 1 describes the clinical characteristics and disease activity of patients, and Figure 2 represents changes of each individual patient’s mSASSS.

**TABLE 1:** Clinical characteristics of 1,125 patients with ankylosing spondylitis (AS).

Variables	All patients
Number, *n*	1,125
Female, *n* (%)	31.97 (9.40)
Age at diagnosis (mean [SD]), years	130 (11.6)
Ocular involvement, *n* (%)	363 (38.3)
Peripheral involvement, *n* (%)	401 (42.8)
HLA B27 positivity, *n* (%)	1,081 (96.5)
**Smoking, *n* (%)**	
Non-smoker	416 (38.8)
Ex-smoker	307 (28.7)
Smoker	348 (32.5)
Baseline BASDAI (mean [SD])	5.12 (2.69)
Baseline BASDAI (median [IQR])	5.45 [2.50, 7.20]
Baseline ESR (mean [SD]), mm/hr	33.17 (31.31)
Baseline ESR (median [IQR]), mm/hr	23.00 [8.00, 48.00]
Baseline CRP (mean (SD)), mg/dL	2.26 (2.59)
Baseline CRP (median [IQR]), mg/dL	1.10 [0.80, 2.60]

SD, standard deviation; HLA, human leukocyte antigen; BASDAI, Bath ankylosing spondylitis disease activity index; IQR, interquartile range; ESR, erythrocyte sedimentation rate; CRP, C-reactive protein.

### Identification of trajectories

**FIGURE 3:**
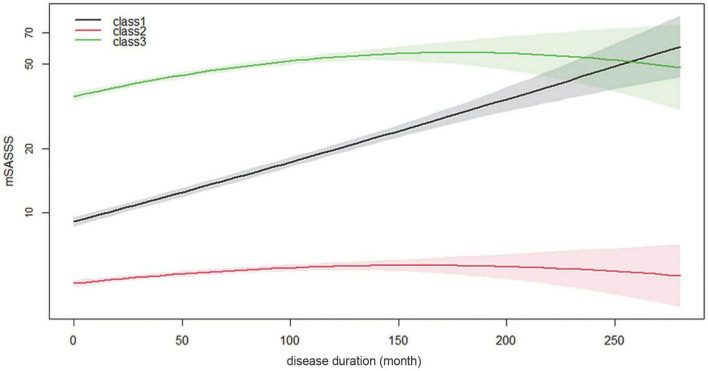
Longitudinal mSASSS trajectory groups for disease duration. Time in month is shown along the x-axis, and logarithmic transformed total mSASSS is shown along the y-axis. The solid line represents the estimated mean in the same-colored area representing the 95% confidence interval. mSASSS, modified Stoke Ankylosing Spondylitis Spinal Score.

In the GBTM analysis, three was determined to be the optimal number of trajectory groups (Supplementary Table 1). Additionally, the average posterior probability was 0.956. This far surpassed the recommended value of 0.7, suggesting that the model had a good fit and that the OCC of the groups was high (110.2). The percentage of patients in the smallest group was calculated as 24.4% (>10%), and the smallest group consisted of approximately 270 patients, which was the most appropriate number to analyze with three trajectories. The trajectories were analyzed by logarithmic transformation of the total mSASSS, and all three groups had distinctly different shapes. The curve/quadratic function was significant in all trajectory groups with an adequate number of patients in each class (Figure 3). Group 1 had 322 patients and a trajectory showing a uniformly increasing slope over the entire period. The change in mSASSS compared to that at the time of diagnosis was the largest in class 1, suggesting a rather rapid progression. Class 2 included 529 patients and showed little change in slope, and thus was considered the non-progression group. Class 3 included 274 patients; this group had the highest mSASSS at the time of diagnosis, but the progression after diagnosis was minimal and thus was considered the early stage increased progression group.

The trajectories according to the identified disease duration showed statistically significant differences in the parameters of sex, age at diagnosis, ocular involvement, peripheral joint involvement, smoking, baseline ESR (mean [SD] and median), log baseline ESR, baseline CRP (median), and log baseline CRP (Table 2). In class 3, the number of male patients, frequency of ocular involvement, and frequency of patients with a smoking history were the highest in each category. In class 2, the number of female patients, frequency of peripheral joint involvement, and number of non-smokers were the highest in each category. In class 1, values related to baseline ESR and CRP levels were the highest among the three classes.

**TABLE 2:** Comparison of trajectory group membership in the three-class mSASSS model for disease duration.

Variables	Class 1	Class 2	Class 3	*P*-value
Number, *n*	322	529	274	
Baseline mSASSS (mean [SD])	**9.84 (3.65)**	**5.17 (1.95)**	**38.33 (17.44)**	**<0.001**
Female, *n* (%)	**39 (12.1)**	**81 (15.3)**	**10 (3.6)**	**<0.001**
Age at diagnosis (mean [SD]), years	**30.91 (9.00)**	**29.10 (8.38)**	**38.74 (8.36)**	**<0.001**
Ocular involvement, *n* (%)	**106 (39.1)**	**144 (32.2)**	**113 (49.1)**	**<0.001**
Peripheral involvement, *n* (%)	**103 (38.9)**	**233 (52.5)**	**65 (28.4)**	**<0.001**
HLA B27 positivity, *n* (%)	307 (96.2)	504 (95.6)	270 (98.5)	0.099
Smoking, *n* (%)				**<0.001**
Non-smoker	119 (38.5)	236 (47.6)	61 (22.9)	
Ex-smoker	91 (29.4)	115 (23.2)	101 (38.0)	
Smoker	99 (32.0)	145 (29.2)	104 (39.1)	
Baseline BASDAI (mean [SD])	4.97 (2.93)	5.11 (2.59)	5.31 (2.63)	0.759
Baseline ESR (mean [SD]), mm/hr	**36.94 (31.61)**	**30.56 (32.26)**	**33.63 (28.65)**	**0.021**
Baseline ESR (median [IQR]), mm/hr	**30.00 [11.00, 54.00]**	**19.00 [4.00, 46.00]**	**26.00 [12.00, 45.00]**	**<0.001**
log baseline ESR (mean [SD]), mm/hr	**3.10 (1.18)**	**2.69 (1.36)**	**3.10 (1.03)**	**<0.001**
Baseline CRP (mean (SD)), mg/dL	2.45 (2.55)	2.20 (2.78)	2.14 (2.25)	0.291
Baseline CRP (median [IQR]), mg/dL	**1.50 [0.80, 3.25]**	**0.80 [0.80, 2.40]**	**1.44 [0.80, 2.20]**	**<0.001**
log baseline CRP (mean [SD]), mg/dL	**0.55 (0.78)**	**0.36 (0.81)**	**0.46 (0.70)**	**0.004**

Bold text means statistically significant values.

mSASSS, modified stoke ankylosing spondylitis spinal score; SD, standard deviation; HLA, human leukocyte antigen; BASDAI, Bath ankylosing spondylitis disease activity index; ESR, erythrocyte sedimentation rate; IQR, interquartile range; CRP, C-reactive protein.

### Associated factors of mSASSS trajectories according to disease progression

We performed a logistic regression analysis to confirm predictors between the trajectories. In class 2 disease progression was predicted to be slow, while in classes 1 and 3 it was predicted to be rapid regardless of combined and analyzed disease duration (Table 3). Statistically significant values were shown for sex, age at diagnosis, ocular involvement, peripheral joint involvement, and log baseline ESR. A higher ratio of female sex was associated with a higher probability of belonging to class 2 (OR = 2.412, 95% CI = 1.381, 4.213), and an older age at diagnosis was associated with a lower probability of belonging to class 2 (OR = 0.505, 95% CI = 0.418, 0.610). If the patient had a history of ocular involvement, the probability of belonging to class 2 was low (OR = 0.607, 95% CI = 0.439, 0.840), while if patient also had a history of peripheral joint involvement, the probability of belonging to class 2 was higher (OR = 2.558, 95% CI = 1.859, 3.520). Furthermore, a higher log baseline ESR was associated with a lower probability of belonging to class 2 (OR = 0.778, 95% CI = 0.685, 0.883). Therefore, the following factors were related to the trajectory according to disease duration for which increased progression was predicted: male sex, younger age at diagnosis, ocular involvement, and high baseline ESR.

**TABLE 3:** Multivariate logistic regression analysis for associated factors of mSASSS progression according to disease duration.

	Class 2 vs. Class 1 and 3							
Variables	Univariate				Multivariate			
	OR	OR 95% CI		*P*-value	OR	OR 95% CI		*P*-value
		Lower	Upper			Lower	Upper	
Sex	2.018	1.386	2.94	<0.001	**2.412**	**1.381**	**4.213**	**0.002**
Age at diagnosis (10y)	0.500	0.432	0.580	<0.001	**0.505**	**0.418**	**0.610**	**<0.001**
Ocular involvement	0.612	0.469	0.798	<0.001	**0.607**	**0.439**	**0.840**	**0.003**
Peripheral involvement	2.143	1.647	2.788	<0.001	**2.558**	**1.859**	**3.520**	**<0.001**
HLA B27 positivity	0.608	0.317	1.163	0.133				
Smoking*				<0.001				0.444
Ex-smoker	0.457	0.338	0.618	<0.001	0.775	0.517	1.163	0.218
Smoker	0.545	0.408	0.727	<0.001	0.836	0.569	1.230	0.364
Baseline BASDAI	0.997	0.911	1.091	0.95				
Baseline ESR	0.995	0.991	0.999	0.013				
Log baseline ESR	0.767	0.694	0.849	<0.001	**0.778**	**0.685**	**0.883**	** < 0.001**
Baseline CRP	0.983	0.937	1.031	0.476				
Log baseline CRP	0.778	0.662	0.913	0.002	^¶^	^¶^	^¶^	^¶^

Bold text means statistically significant values.

*Analysis with non-smoker as a reference.

^¶^It was excluded when performing multivariate analysis due to multicollinearity.

mSASSS, modified stoke ankylosing spondylitis spinal score; OR, odds ratio; CI, confidence interval; HLA, human leukocyte antigen; BASDAI, Bath ankylosing spondylitis disease activity index; ESR, erythrocyte sedimentation rate; CRP, C-reactive protein.

As classes 1 and 3 showed different trajectories, they were comparatively analyzed (Table 4), with statistical significance estimated for sex, age at diagnosis, ocular involvement, and peripheral joint involvement. According to the analysis, female patients were less likely to belong to class 3 (OR = 0.141, 95% CI = 0.049, 0.401), and a higher age at diagnosis was associated with a higher probability of belonging to class 3 (OR = 3.378, 95% CI = 2.549, 4.478). If the patient had a history of ocular involvement, the probability of belonging to class 3 was high (OR 1.676, 95% CI [1.096, 2.561]), while if the patient had a history of peripheral joint involvement, the probability was lower (OR = 0.621, 95% CI = 0.395, 0.979). Therefore, for a male patient or a patient with history of ocular involvement, spinal lesions would be expected to progress rapidly at initial diagnosis (class 3).

**TABLE 4:** Multivariate logistic regression analysis of mSASSS progression for associated factors in class 3 and class 1 according to disease duration.

	Class 3 vs. Class 1							
Variables	Univariate				Multivariate			
	OR	OR 95% CI		*P*-value	OR	OR 95% CI		*P*-value
		Lower	Upper			Lower	Upper	
Sex	0.275	0.135	0.562	<0.001	**0.141**	**0.049**	**0.401**	**<0.001**
Age at diagnosis (10y)	2.856	2.277	3.583	<0.001	**3.378**	**2.549**	**4.478**	**<0.001**
Ocular involvement	1.503	1.054	2.145	0.025	**1.676**	**1.096**	**2.561**	**0.017**
Peripheral involvement	0.623	0.427	0.911	0.014	**0.621**	**0.395**	**0.979**	**0.040**
HLA B27 positivity	2.638	0.841	8.277	0.096				
Smoking*				<0.001				0.833
Ex-smoker	2.165	1.424	3.292	<0.001	1.091	0.619	1.921	0.764
Smoker	2.049	1.355	3.099	0.001	1.186	0.678	2.075	0.551
Baseline BASDAI	1.045	0.926	1.18	0.473				
Baseline ESR	0.996	0.991	1.002	0.201				
Log baseline ESR	0.995	0.855	1.157	0.947				
Baseline CRP	0.946	0.88	1.018	0.138				
Log baseline CRP	0.851	0.678	1.068	0.164				

Bold text means statistically significant values.

*Analysis with non-smoker as a reference.

mSASSS, modified stoke ankylosing spondylitis spinal score; OR, odds ratio; CI, confidence interval; HLA, human leukocyte antigen; BASDAI, Bath ankylosing spondylitis disease activity index; ESR, erythrocyte sedimentation rate; CRP, C-reactive protein.

### Decision tree analysis of each class of mSASSS trajectories

**FIGURE 4:**
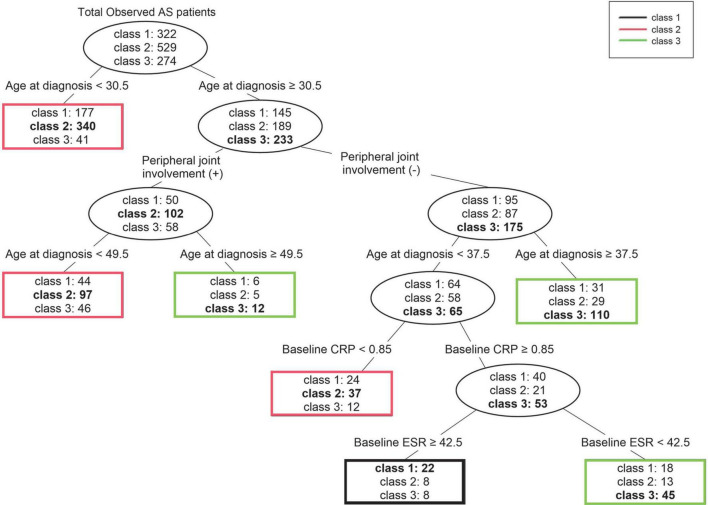
Decision tree for prediction of three trajectory classes according to statistically significant clinical factors. For each terminal node, the class written in bold is the class that represents that node. AS, ankylosing spondylitis; CRP, C-reactive protein; ESR, erythrocyte sedimentation rate.

The same input variables used for logistic regression analysis were also used in the decision tree analysis (Figure 4). For the whole data set, age was the first splitting variable. For a cutoff of age <30.5 years, the purity of node age <30.5 years could not be improved; thus, division was finished and this node became terminal, represented as class 2. The second splitting was determined for the presence of peripheral joint involvement. The next splitting was once again based on age. For the node with peripheral joint involvement the cutoff was 49.5 years, and for the node without peripheral joint involvement the cutoff was set at 37.5 years. The nodes with peripheral joint involvement were highly likely to be classified as class 2 when younger than 49.5 years and class 3 when older than 49.5 years of age. The nodes without peripheral joint involvement were more likely to be classified as class 3 when older than 37.5 years of age. Since all three nodes could no longer be improved in purity, the splitting was finished, and the division continued if age was less than 37.5 years and there was no peripheral joint involvement. The fourth splitting was based on baseline CRP (cutoff 0.85 mg/dL), and when the CRP was lower than the cutoff a terminal node classified as class 2 was formed. Finally, at a node with a CRP higher than the cutoff splitting was carried out based on the baseline ESR (cutoff 42.5 mm/hr), and a terminal node with a node higher than the cutoff of class 1 and a node lower than the cutoff of class 3 were formed. The accuracy (proportion of correctly classified patients) of our decision tree was 58.9% (Supplementary Table 2).

## Discussion

The purpose of this study was to identify changes in radiographic progression patterns and to predict radiographic progression using the derived associated factors from 18 years of real-world data of patients with AS. We analyzed changes in mSASSS according to temporal changes in disease duration and classified them into three groups with distinctly different patterns. Through prediction using a decision tree by that applied clinical factors to three classified trajectories, older age at diagnosis and ocular involvement were shown to be more associated with the group showing high mSASSS during the disease period (class 3), while younger age at diagnosis and peripheral joint involvement were more associated with the group that maintained low mSASSS, or class 2. Although these associated factors are already known (1–3, 34), smoking, known to be strong risk factor for disease progression of AS (8–14), did not act as a related factor for each trajectory (2).

We found the most notable result of the trajectories to be the difference between classes 1 and 3. In particular, class 1 showed less radiographic progression at initial diagnosis, yet had a pattern of increasing mSASSS as the disease period increased. Class 3 showed increased radiographic progression at initial diagnosis than class 1. However, after 20 years or more disease progression, class 1 was predicted to have mSASSS similar to or higher than that of class 3. In the predictions made through the decision tree, for patients aged 30.5 years or older at time of diagnosis and lacking peripheral joint involvement, and when age at diagnosis was less than 37.5 years and baseline CRP was 0.85 mg/dL or more, the baseline ESR was higher (above 42.5 mm/hr) and these patients were more likely to belong to class 1. Therefore, among patients diagnosed in their third decade and lacking peripheral joint involvement, patients with high acute-phase reactant could be predicted to show a radiographic progression pattern in which mSASSS continues to increase, even if mSASSS was not high at diagnosis. These findings may help clinicians predict a patient’s radiographic progression in advance so that the appropriate treatment plan can be established.

Among recent studies, there was a study that was conducted with a purpose similar to our’s (27). Hwang et al. had a similar goal of predicting radiographic changes over time, and they analyzed 561 patients divided into four distinct groups. Since they used data from an already formed cohort, the disease status prior to enrollment in the cohort was not reflected. We conducted the study with a larger number of patients (1,125), and the disease status from the time of diagnosis was reflected in all patients. Since there was no additional selection process other than the number of radiographs on the patient, selection bias was minimized. Further, due to the characteristics of trajectory analysis, a meaningful finding cannot be confirmed only with the obtained trajectory; therefore, we additionally introduced a decision tree for predicting trajectory to help clinical practice. However, compared to our study, it is significant that they further identified a response to the use of drugs such as tumor necrosis factor inhibitor (TNFi). They suggested that only patients in the “rapid-progressor” group reduced changes in mSASSS to TNFi drugs, which is considered a very noteworthy clinical feature, which will be a good reference for planning our follow-up studies.

There were some limitations to this study. First, the accuracy of the trajectories was not guaranteed, as the prespecified rule did not provide a method for correcting accuracy of individual classifications for the various groups constituting the classes. Therefore, if the observation period, number of included patients, or clinical characteristic were to change, the number and pattern of trajectories may also change. Second, trajectory changes occurred as a result of the limitations of the analysis method and its accuracy. In Figure 3, the trajectory of class 3 indicates that the mSASSS decreased slightly as time passed, likely owing to a small or non-existent number of patients with long disease duration. We considered these two limitations of the GBTM method and concluded that it was clinically and statistically most appropriate to analyze the three trajectories, and we found and presented the best trajectory to help clinicians make decisions about treatment and prognosis. The trajectories of mSASSS may have also differed if the observation period had been longer or more patients had been included. Additionally, the accuracy of the decision tree in this study was low; however, this was a result of the involvement of numerous clinical factors, which was a limitation of the decision tree analysis method itself (35, 36). Thus, since our purpose was to develop a predictive model, we applied this method. Lastly, most of the factors analyzed as predictors of trajectory were already well-known factors. However, the purpose of this study was to find a way to predict progression rather than to clarify “factors,” and to analyze it including the concept of time course. In particular, since progression of the spine is largely irreversible, its prediction is important for both patients and clinicians. We thought that understanding the changing patterns of spinal progression in AS and identifying the clinical factors associated with it could provide new insights that could clarify treatment goals for different subgroups of patients with AS.

## Conclusion

This study identified three groups with different radiographic progression patterns and predicted changes in these patterns. We found the predictions to be affected by the factors of sex, age at diagnosis, ocular involvement, peripheral joint involvement, and acute-phase reactant level. The model developed herein offers the useful clinical application of predicting the radiographic progression of the spine in patients with AS, predicting the course of the disease. Thus, it may thus aid in decisions regarding timing of treatment interventions in patients with specific characteristics.

## Data availability statement

The original contributions presented in this study are included in the article/Supplementary material, further inquiries can be directed to the corresponding author/s.

## Ethics statement

The studies involving human participants were reviewed and approved by Institutional Review Board of Hanyang University Hospital. Written informed consent for participation was not required for this study in accordance with the national legislation and the institutional requirements.

## Author contributions

T-HK and BK had full access to all data in the study. SP was responsible for data analysis and ensured the accuracy of data analysis. SL contributed to the acquisition and accuracy of image data. JK and T-HL contributed to the research concept and design. All authors contributed to the collection, analysis, and interpretation of the data. JK, BK, and T-HK drafted the manuscript and contributed mainly to the interpretation of the data. All authors have read and accepted the final manuscript.
