# Supplementary material for: Prediction of radiographic progression pattern in patients with ankylosing spondylitis using group-based trajectory modeling and decision trees
Source: Front Med (Lausanne). 2022 Oct 20;9:994308. doi: 10.3389/fmed.2022.994308 (PMC9631932; doi:10.3389/fmed.2022.994308)
Supplement: Supplementary file 1 [file Table_1.docx]

**Supplementary table 1.** Model fit for group-based trajectory modelling of mSASSS according to disease duration

| **Number of groups** | **AIC** | **BIC** | **APPA** | **OCC** | **% patient in the smallest group** |
| --- | --- | --- | --- | --- | --- |
| 2 | 9774.1 | 9814.3 | 0.988 | 84.5 | 34.7 |
| **3** | **8256.2** | **8316.6** | **0.956** | **110.2** | **24.4** |
| 4 | 6720.6 | 6801.0 | 0.968 | 132.0 | 7.8 |
| 5 | 6018.6 | 6119.1 | 0.939 | 377.1 | 4.6 |
| 6 | 5487.4 | 5608.0 | 0.945 | 294.5 | 4.5 |

Note: Bold text means that the most appropriate number of models was adopted considering all values.
AIC, Akaike information criteria; BIC, Nagin Bayesian information criteria; APPA, average posterior probability of assignment; OCC, odds of correct classification.
