# Supplementary material for: Prediction of radiographic progression pattern in patients with ankylosing spondylitis using group-based trajectory modeling and decision trees
Source: Front Med (Lausanne). 2022 Oct 20;9:994308. doi: 10.3389/fmed.2022.994308 (PMC9631932; doi:10.3389/fmed.2022.994308)
Supplement: Supplementary file 2 [file Table_2.docx]

**Supplementary table 2.** Predicted classes by tree analysis of three trajectory groups

|  | **Class 1** (n=322) | **Class 2**  (n=529) | **Class 3**  (n=274) |
| --- | --- | --- | --- |
| Predicted Class 1 | **22** | 8 | 8 |
| Predicted Class 2 | 245 | **474** | 99 |
| Predicted Class 3 | 55 | 47 | **167** |
| Accuracy (proportion of correctly classified patients) = 58.9% | | | |

Note: Text in bold is when the predicted class is a match predicted to be the true class.
